# Exploring the Sex-Differentiated Transcription of GnRH1/GnRHR1 Signaling in Hamster

**DOI:** 10.3390/life16040620

**Published:** 2026-04-08

**Authors:** Aidet Ruiz, Luis Ramos

**Affiliations:** Department of Reproductive Biology, Instituto Nacional de Ciencias Médicas y Nutrición Salvador Zubirán, México City 14080, Mexico; aidet.ruizg@incmnsz.mx

**Keywords:** GnRH1, GnRHR1, neuropeptides, reproduction, endocrinology

## Abstract

Gonadotropin-releasing hormone 1 (GnRH1) and its receptor (GnRHR1) are central neuropeptides on the hypothalamic–hypophysis–gonadal (HHG) axis and play key roles in vertebrate reproduction. Although GnRH1/GnRHR1 signaling has been extensively studied in models such as mouse, rat, zebrafish, and human, knowledge from other species is limited. This work used cloning, Sanger sequencing, and qPCR to highlight the molecular structure, evolutionary history, and sex-differentiated transcription of GnRH1/GnRHR1 signaling from hamster. These findings showed that GnRH1/GnRHR1 hamster proteins exhibit a molecular evolutionary history highly similar for peptides reported in other species and with which they share a high degree of structural homology. Expression profiles indicated a GnRH1 transcript in several tissues with higher expression levels in testes, adrenals, uterus, epididymis, female hypothalamus, and Harderian glands. GnRHR1 expression levels were seen exclusively in male and female hypophysis with higher levels in female hypophysis. Expression levels showed significant differences for GnRH1 in several tissues during estrous; GnRHR1 expression during estrous was detected only in hypophysis with increased expression levels seen during metestrus and diestrus. These results suggest a highly conserved homology of GnRHR1/GnRHR1 signaling, thus highlighting its evolutionary importance. These expression levels underscore the importance of GnRHR1 as a master regulator of reproductive endocrinology and could implicate hamster peptides as potential therapeutic biological models for human endocrine diseases.

## 1. Introduction

The sexual and reproductive health of both female and male mammalian/non-mammalian vertebrates is regulated by the hypothalamic–hypophysis–gonadal (HHG) axis [1,2]. This axis is primarily activated by the hypothalamic gonadotropin-releasing hormone (GnRH1) and controlled by the GnRH receptor (GnRHR1). This cell-surface receptor can mediate actions via association with G-proteins and can activate a phosphatidylinositol-calcium second messenger system to initiate cellular signaling pathways that stimulate the synthesis and secretion of the gonadotropins [3,4,5], follicle-stimulating hormone (FSH), and luteinizing hormone (LH) that drive gonadal function, steroidogenesis, and sexual behavior [6,7]. In humans, the HHG axis is critical for expression of sexual characteristics. Dysregulation of this axis leads to clinical and pathological characteristics associated with reproductive disorders, such as congenital hypogonadotropic hypogonadism (CHH) and delayed puberty—a rare genetic disorder caused by a defect in GnRH1secretion by the hypothalamus or a defect in action of GnRHR1 on the pituitary gland. CHH is an inherited endocrine disorder characterized by cryptorchidism, micropenis, and infertility in males. Females see limited primary amenorrhea as well as failure to go through puberty, growth retardation, and infertility in females [8,9,10].

The GnRH1/GnRHR1 signaling pathway is a key endocrine mechanism of the HHG axis [11]. Although its primary action occurs in central endocrine physiology, studies in various biological species have shown that both GnRH1 and GnRHR1 are expressed in multiple extrahypothalamic or extrahypophyseal peripheral tissues suggesting local autocrine and paracrine functions in the regulation of sexual and reproductive health, sexual behavior, and the development of secondary sexual characteristics [12,13]. In mammals, including humans, GnRH1 expression has been reported primarily in the hypothalamus, preoptic nucleus, gonads, uterus, and placenta. In birds and teleost fish, its expression has been observed in the hypothalamus, midbrain, medulla oblongata, and gonads. Similarly, in different vertebrates, the predominant sites of GnRHR1 expression have been identified in the pituitary gland, brain, gonads, uterus, endometrium, and placenta. In vertebrates, the expression of more than one form or molecular variant of the neuropeptide GnRH (GnRH1, GnRH2, and GnRH3) and its receptor GnRHR (GnRHR1, GnRHR2, and GnRHR3—all encoded by different genes) has been described. All GnRHs share the ability to activate GnRHR. Like GnRHR, they differ in sequence, evolutionary origin, distribution, and physiological function. GnRH1/GnRHR1 is the main regulatory signaling pathway for reproduction and is present in most vertebrates, specifically mammals [14,15,16].

As a result, studying the molecular characterization and transcription patterns of the GnRH1/GnRHR1 signaling pathway is an important step to further understand reproductive endocrinology and intracrinology of the signaling pathway. However, further studies are needed to unveil the evolutionary diversity of the GnRH1/GnRHR1 system, investigate the non-reproductive roles of the GnRH1/GnRHR1 signaling pathway, identify the extrahypothalamic GnRH1/GnRHR1 system, analyze its autocrine/paracrine function, and study the expression of the multiple non-classical variants of GnRH1/GnRHR1 signaling and their functions. Previous reports have explored biological models to reveal the molecular mechanisms of reproductive signaling [17] such as the hamster (*Mesocricetus auratus*). Some of biological and reproductive characteristics that led to the selection of hamster as a model species for studying the GnRH1/GnRHR1 signaling pathway include its well-defined seasonal reproductive cycles. Some tissues such as the Harderian gland (HG) exhibit marked sexual dimorphism controlled by sex steroids [18,19,20], protein hormones, as well as gene regulation mediated by sex steroids and their reproductive effects [21,22]. Previous studies have demonstrated sex differences influenced by pituitary and gonadal hormones that make the hamster a more promising research subject than other rodents; these differences include variations in porphyrinogenic activity, the number of mast cells, activity related to melatonin synthesis, and 5α-steroid reductase activity. Therefore, the purpose of this research was to understand how hormonal signaling via GnRH1/GnRHR1 is expressed in the HHG axis and to demonstrate the originality of this biological model. In this study, the molecular architecture and transcriptional profiles of the GnRH1/GnRHR1 signaling pathway in multiple hamster tissues were explored with the aim of comparing and extrapolating findings to other biological species including humans. In addition, these findings are compared with prior results to determine how hamsters could help explain reproduction.

## 2. Material and Methods

### 2.1. Ethics Statement and Hamster Tissues

All animal experimental procedures were performed in accordance with our institution’s ethical committee (INCMNSZ, BRE-1930-18-19-1). The hamsters were offspring of a colony maintained at Universidad Autónoma Metropolitana-Xochimilco (UAM-X; México City, México; code number AUT-B-C-0215–016). Hamsters were individually caged under standard conditions in a temperature and humidity-controlled room on a 12 h light/dark cycle. The calculation of the number of animals required is based on the descriptive nature of the study. The formula used to calculate the sample size for gene expression assays is based on the distribution of qPCR data: n = $n=\frac{2{\left(Z_{\alpha /2}+Z_{\beta }\right)}^{2}\sigma^{2}}{{∆}^{2}}$; where n = sample size per group, Z_(α/2)_ = Z-value for the significance level, Z_β_ = Z-value for statistical power, σ = standard deviation of gene expression, and Δ = fold change in gene expression. Considering, Z_(α/2)_ = Z_(0.05/2)_ = 1.96, Z_β_ = Z_0.8_ = 0.84, σ = 1, and Δ = 2, one can estimate that four biological replicates (i.e., five tissues from different individuals) are required per group to be studied. Five males and five females each in proestrus, estrus, metestrus, and diestrus were used; they all had access to food and water ad libitum. The estrous cycle was determined using smears from exfoliated hamster vaginal cells. Male and female hamsters were anesthetized via intramuscularly injection of ketamine/xylazine (80 mg/kg: 8 mg/kg) and sacrificed via decapitation. Lung, brain, cerebellum, hypothalamus, epididymis, uterus, testis, ovary, hypophysis, adrenal, and HG were isolated from adult hamsters (10 months old; 150–200 g; 6 inches in length) from both sexes. The tissues were placed on dry ice and immediately stored in an ultra-freezer (−70 °C) until use.

### 2.2. RNA Evaluation and cDNA Synthesis

Briefly, 50–100 mg of tissue was isolated and homogenized using 1 mL of TRIzol reagent (Invitrogen, Carlsbad, CA, USA) and a tissue homogenizer in accordance with guideline’s protocol. The total RNA was resuspended in 40–100 μL of diethyl pyrocarbonate (DEPC)-treated distilled water and treated with RNase-free DNase I to remove DNA contamination from total RNA samples. A spectrophotometer (Beckman DU 650, Fullerton, CA, USA) was used at an A_260_/A_280_ ratio of 1.8 to assess the quantity and quality of total RNA; the integrity of each RNA was validated by identifying ribosomal RNA (28S:18S rRNA ratio) using agarose/formaldehyde/MOPS denaturing gel electrophoresis. The total RNAs that met the minimum requirements for quantity, quality, and integrity were used for subsequent cDNA synthesis. Based on the average total RNA observed using spectrophotometry, 2.0 μg total RNA was used for cDNA synthesis from each tissue type using both a Transcriptor First Strand cDNA Synthesis kit for cloning (Roche Diagnostics, Indianapolis, IN, USA) and a Maxima First Strand cDNA Synthesis kit (ThermoScientific, Vilnius, Lithuania) for real-time quantitative PCR (qPCR). The synthesized cDNA was then stored at −80 °C for subsequent experiments.

### 2.3. Characterization of the Transcripts Using 5′- and 3′-RACE

Next, to characterize the GnRH1/GnRHR1 transcripts, 5′- and 3′- rapid amplification of cDNA ends (RACE) was performed using the SMARTer™ RACE cDNA Amplification kit (Takara Bio Inc., Mountain View, CA, USA) according to the manufacturer’s instructions. The existing GnRH1/GnRHR1 sequences were used as a guide to design primers using PrimerQuest™: PCR and qPCR primer design tool (https://www.idtdna.com/page). Four gene-specific primers (GSPs) based on known sequences (*Mus musculus*, *Rattus norvegicus*, and *Homo sapiens*) of conserved regions were designed for RACE (GnRH1: RACE5′ = 5′-gagcagccttccaaacataggctcag-3′; RACE3′ = 5′-ctcagggaccttcgaggagttctgg-3′; GnRHR1: RACE5′ = 5′-cttcctccctttctccttcttctgag-3′; RACE3′ = 5′-atcatcttcaccctcacacgggtcctc-3′). The 5′- and 3′-RACE products were electrophoretically separated in a 1.2% agarose gel with 1X TBE buffer. The resulting bands were recovered with the GeneJET gel extraction kit (Thermo Fisher Scientific, Vilnius, Lithuania) and then sequenced. The partial length sequences were used to design four specific primers (GnRH: 5′-acccaccaaaggaagtttgta-3′, 5′-catgttacacttgggtgttgtg-3′; GnRHR: 5′-aaacatcagcagtaacagagact-3′, 5′-agttatcccgtatatgggtttcag-3′) with subsequent amplification of the full-length cDNA of GnRH1 and GnRHR1.

### 2.4. Recombinant Plasmid Construction and Sanger Sequencing

To sequence GnRH1/GnRHR1, the full-length cDNAs were cloned using a TOPO TA cloning kit for sequencing following the manufacturer’s instructions (Invitrogen Co., Carlsbad, CA, USA) and transformed into competent *Escherichia coli* strain DH5α cells using the heat-shock method. DNA was extracted using the Plasmid DNA Mini kit (Omega Bio-Tek, Inc., Norcross, GA, USA) according to the manufacturer’s instructions. Successfully transformed cells were then grown and purified using the Plasmid DNA Maxi kit (Omega Bio-Tek, Inc., Norcross, GA, USA) following the manufacturer’s recommendation. Either full-length cDNAs cloned in the pCR^®^II-TOPO vector were Sanger-sequenced as described previously in detail [17]. The resulting nucleotide sequences of GnRH1/GnRHR1 were then subjected to a thorough analysis using Chromas Biotechnology software (version 2.6.6).

### 2.5. Elucidation of Peptide Sequences

Amino acid sequences were deduced using the ExPASy (https://web.expasy.org/translate/ (accessed on 6 January 2025)). Protein characterization was performed using ProtParam on ExPASy server (https://www.expasy.org/resources/protparam (accessed on 6 January 2025)) to determine the molecular weight and theoretical isoelectric point of GnRH1/GnRHR1. The blastp (https://blast.ncbi.nlm.nih.gov/Blast.cgi), CLUSTALΩ (https://www.ebi.ac.uk/jdispatcher/msa/clustalo (accessed on 6 January 2025)), and CLUSTALW (https://www.genome.jp/tools-bin/clustalw (accessed on 6 January 2025)) algorithms were used to compare the sequences with GnRH1/GnRHR1 in GenBank (https://www.ncbi.nlm.nih.gov/gene/ (accessed on 6 January 2025)). The prediction of eukaryotic protein subcellular localization used DeepLoc-2.1 (https://services.healthtech.dtu.dk/services/DeepLoc-2.1/ (accessed on 6 January 2025)). The prediction of signal peptides and their cleavage sites used SignalP-6.0 (https://services.healthtech.dtu.dk/services/SignalP-6.0/ (accessed on 6 January 2025)). The prediction of transmembrane helices used TMHMM-1.0 (https://services.healthtech.dtu.dk/service.php?DeepTMHMM-1.0 (accessed on 6 January 2025)). Phylogenetic analysis used Molecular Evolutionary Genetics Analysis (MEGA) 12.1 software (https://www.megasoftware.net/) via the maximum-likelihood phylogenetic trees with 1000 bootstrap replicates; representative sequences were retrieved from the GenBank database. The three-dimensional structures of GnRH1/GnRHR1 proteins were predicted using Robetta software (http://new.robetta.org/). The structures were visualized with the PyMOL 3.1 software (https://www.pymol.org/).

### 2.6. Gene Expression Assessment

Two µg of total RNA was used from different tissues for first-strand cDNA synthesis. After synthesizing first-strand cDNA, quantitative PCR was performed using the Maxima First Strand cDNA Synthesis kit (ThermoScientific, Vilnius, Lithuania) as per the manufacturer’s instructions. The qPCR experiments followed the protocol provided by the manufacturer (LightCycler^®^ 480 Probes Master; Roche Diagnostics, Indianapolis, IN, USA). Transcripts of GnRH1 and GnRHR1 were detected using the universal fluorogenic probes #65 (04688643001; 5′-atgtttggaaggctgctcca-3′ and 5′-gccagctgatccacctcttt-3′) and #21 (04686942001; 5′-tgtctttgcaggaccacagttatata-3′ and 5′-tgtgtcacacattgagagaaaacttc-3′), respectively. The *β-actin* (*ACTB*) gene (probe #9 04685075001; 5′-agctatgagctgcctgatgg-3′ and 5′-caggaaggaaggctggaaa-3′) was used as the internal reference. The *GnRH1*/*GnRHR1* gene expression values were detected on a LightCycler 480 instrument (Roche Diagnostics, Indianapolis, IN, USA). Five independent replicates were used for each qPCR tissue, and the relative expression levels were normalized using the 2^−ΔΔCt^ method.

### 2.7. Quantitative Analysis

Data analysis was conducted from the mean and standard deviation (SD) of five hamsters per group. Statistical analysis used GraphPad Prism Software v8.0 (GraphPad Software, San Diego, CA, USA). Means comparisons under different physiological conditions were performed using the two-way analysis of variance (ANOVA). *p*-values < 0.05 were considered statistically significant. Additionally, an ANOVA with a post hoc Sidak test was conducted to compare means between different tissues in male and female hamsters. Data were also evaluated with post hoc Tukey tests during the estrous cycle.

## 3. Results

### 3.1. Sanger Sequencing Analysis

**Figure 1:**
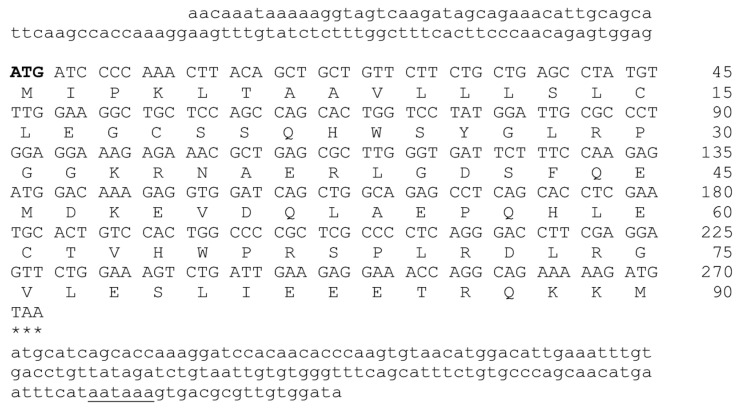
Nucleotide sequence and amino acid sequence of *M. auratus* GnRH1. The start codon (ATG) of ORF is indicated in bold font, and the stop codon (TAA) at the end of the ORF is marked with three asterisks. Signal polyadenylation (AATAAA) is underlined. The 5′- and 3′-UTRs are shown in lowercase letters. The numbers indicate the positions in nucleotide and amino acid sequence.

**Figure 2:**
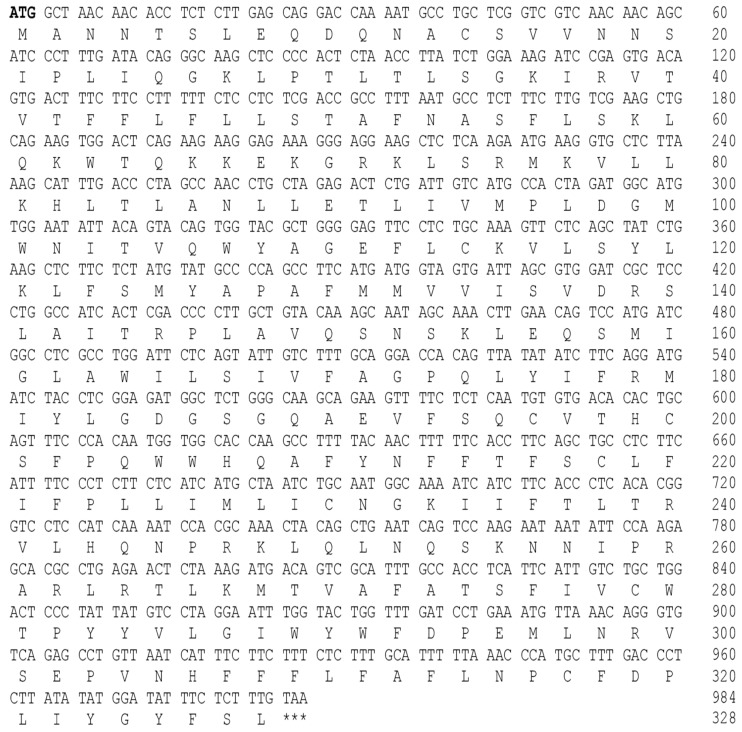
Nucleotide sequence and amino acid sequence of *M. auratus* GnRHR1. The start codon (ATG) of ORF is indicated in bold font, and the stop codon (TAA) at the end of the ORF is marked with three asterisks. The numbers indicate the positions in nucleotide and amino acid sequence. The 5′- and -3′ UTRs of the *M. auratus* GnRHR1 were not isolated or identified.

Nucleotide sequences were isolated and amino acid sequences were deduced to explore the molecular structure of GnRH1/GnRHR1. The full-length cDNAs of GnRH1 (GenBank accession number PX873585)/GnRHR1 (GenBank accession number PX873586) identified in *M. auratus* are 518 and 987 base pairs (bp) long and encode polypeptides of 90 (Figure 1) and 328 (Figure 2) amino acid residues with a predicted molecular weight of 10.27 and 37.87 kDa as well as a theoretical isoelectric point of 5.89 and 9.57, respectively. The full-length cDNA of GnRH1 comprised a 100 bp 5′-untranslated region (UTR) and a 146 bp 3′-UTR with a canonical polyadenylation signal AATAAA; the 5′- and 3′-UTR were absent in full-length cDNA of GnRHR1.

### 3.2. Protein Sequences Homologous

**Figure 3:**
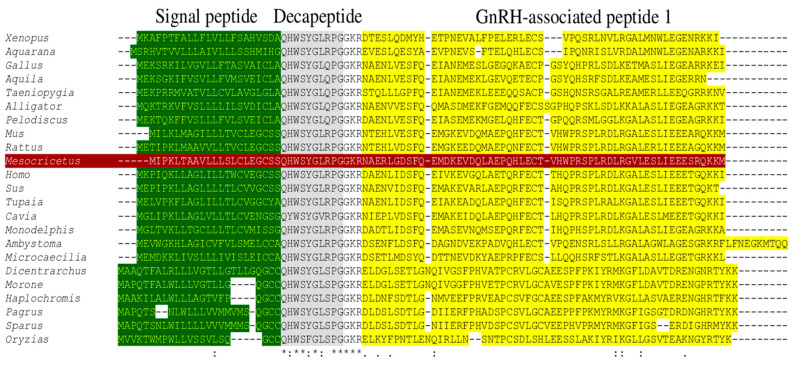
The amino acid sequence of GnRH1 protein and multiple sequence alignment from *M. auratus* and different species. The signal peptide sequence is indicated in green. The decapeptide conserved of GnRH1 is indicated in grey. The GnRH-associated peptide 1 is show in yellow. The amino acid sequence of GnRH1 protein from *M. auratus* is indicated in red. *Mus musculus*, *Rattus norvegicus*, *Tupaia belangeri*, *Homo sapiens*, *Sus scrofa*, *Cavia porcellus*, *Gallus gallus*, *Xenopus laevis*, *Aquarana catesbeiana*, *Haplochromis burtoni*, *Pagrus major*, *Sparus aurata*, *Dicentrarchus labrax*, *Morone saxatilis*, *Oryzias latipes*, *Alligator mississippiensis*, *Pelodiscus sinensis*, *Taeniopygia guttata*, *Aquila chrysaetos chrysaetos*, *Ambystoma mexicanum*, *Microcaecilia unicolor*, *Monodelphis domestica*. The identical, conservative and highly conservative amino acids are illustrated in (*), (.), and (:), respectively.

**Figure 4:**
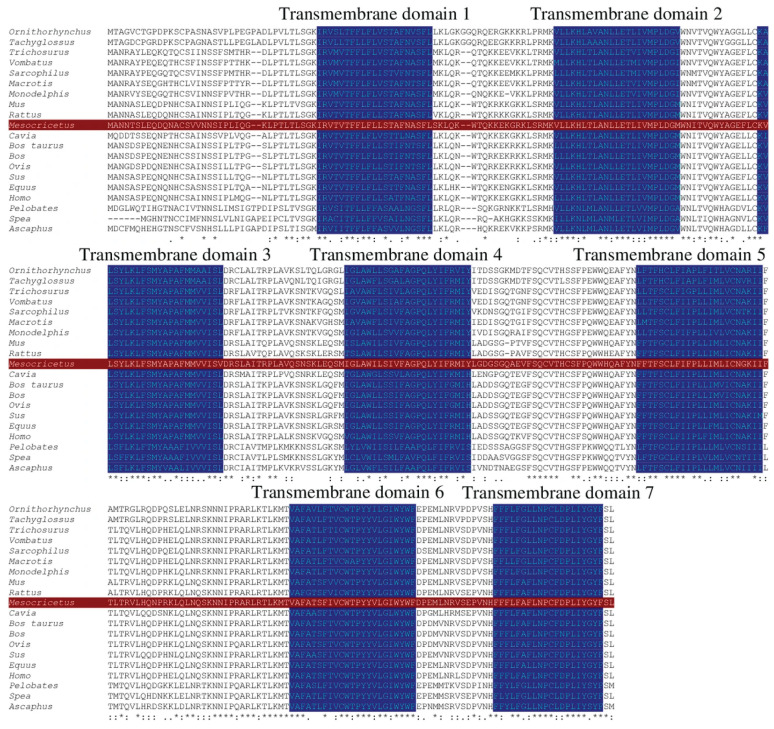
Amino acid sequence, conserved motif analysis and multiple sequence alignment of *M. auratus* and vertebrate GnRHR1. The seven transmembrane domains and conserved sites are labeled with blue. The amino acid sequence of GnRH1 protein from *M. auratus* is indicated in red. *Mus musculus*, *Rattus norvegicus*, *Homo sapiens, Equus caballus*, *Sus scrofa*, *Bos taurus*, *Bos grunniens, Ovis aries*, *Cavia porcellus*, *Trichosurus Vulpecula*, *Ornithorhynchus anatinus*, *Monodelphis domestica*, *Pelobates fuscus*, *Spea bombifrons*, *Ascaphus truei*, *Vombatus ursinus*, *Tachyglossus aculeatus*, *Sarcophilus harrisii*, *Macrotis lagotis*. The identical, conservative and highly conservative amino acids are illustrated in (*), (.), and (:), respectively.

A signal peptide was identified between 21 and 22 amino acids (probability 0.974691) to identify the domains and use bioinformatic tools; no transmembrane domain was identified in the GnRH1 protein. Comparative analysis identified three important domains for hamster GnRH1: a signal peptide located at amino acids 1–21, a mature decapeptide between amino acids 22–31, and a GnRH-associated peptide 1 region between amino acids 35–90. (Figure 3). No signal peptide was identified in GnRHR1 protein; structural predictions identified seven transmembrane domains between 37–57, 78–100, 115–137, 160–182, 213–235, 270–292, and 307–326 amino acids (Figure 4).

### 3.3. Phylogenetic Analysis

**Figure 5:**
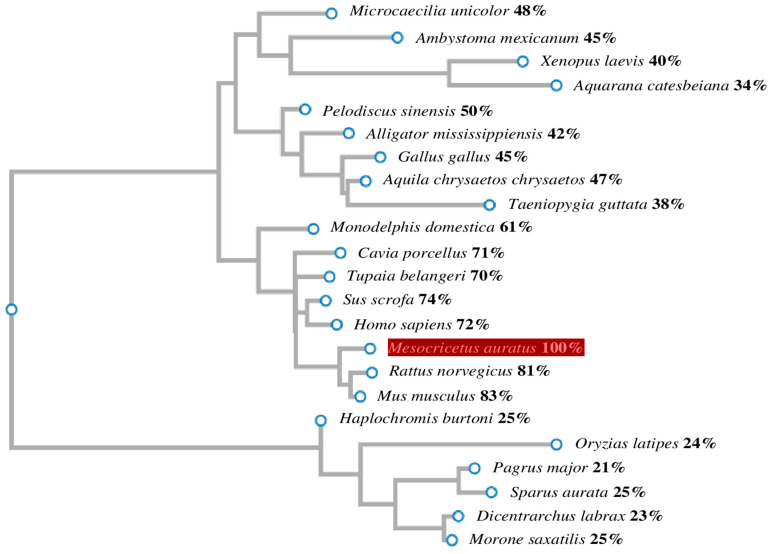
Phylogenetic analysis of GnRH1 protein. The phylogenetic tree shows the evolutionary relationship of the amino acid GnRH1 sequence. The percentages of identity between vertebrate groups are shown on the right. The phylogenetic trees revealed that GnRH1 of *M. auratus* clustered with 22 vertebrates. Phylogeny of 22 GnRH1 proteins was determined by MEGA-X, CLUSTALW, and CLUSTALΩ programs. The amino acid sequence of GnRH1 protein from *M. auratus* is indicated in red.

**Figure 6:**
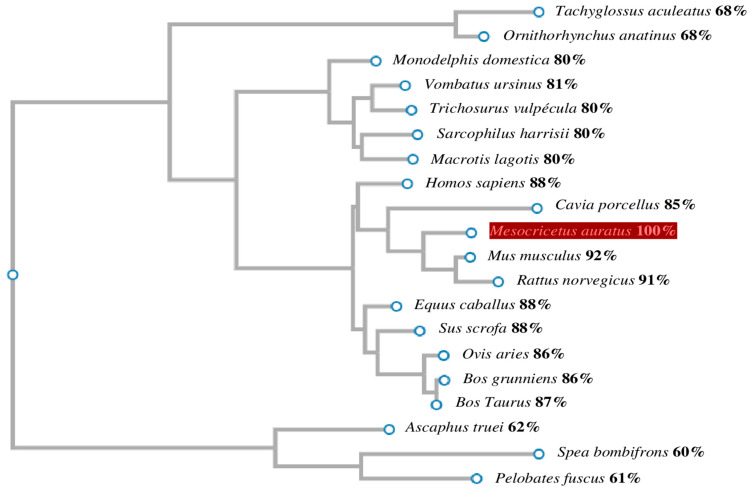
Nineteen GnRHR1 proteins were used for phylogenetic analysis by MEGA-X, CLUSTALW, and CLUSTALΩ programs. The tree branched the GnRHR1 proteins into two subgroups based on the homology of *M. auratus*, which are represented by clusters of different vertebrates within anurans and mammals. The identity between amino acid sequences of the GnRHR1 proteins is indicated as a percentage on the right side. The amino acid sequence of GnRH1 protein from *M. auratus* is indicated in red.

We next designed phylogenetic trees from amino acid sequences to better understand the evolution of the GnRH1/GnRHR1 families. Phylogenetic trees were constructed based on a comparative analysis of *M. auratus* and other species revealing that GnRH1/GnRHR1 share a clade with vertebrates. The amino acid sequence identity of *M. auratus* GnRH1 ranges from 21–83% when compared with other GnRH1 proteins (Figure 5). The amino acid sequence of *M. auratus* GnRHR1 showed a range of identity with other transmembrane receptors for GnRH1 in a range of 60–92% identity (Figure 6). In particular, the analysis revealed that GnRH1 from *M. auratus* is a protein closely related to fish, amphibians, reptiles, birds, and mammals; GnRHR1 from *M. auratus* exhibited a more specific and exclusive relationship with anurans and mammals.

### 3.4. Three-Dimensional Architecture

**Figure 7:**
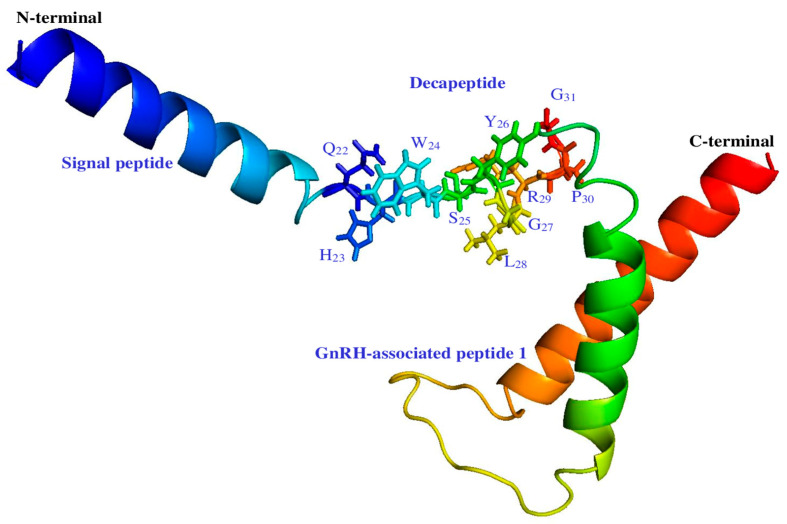
Robetta and PyMOL software visualized the three-dimensional configuration of *M. auratus* GnRH1. Structural representation showing the N-terminal domain, signal peptide, decapeptide, GnRH-associated peptide 1, and C-terminal domain. The stick visualization shows the decapeptide (Q_22_-H_23_-W_24_-S_25_-Y_26_-G_27_-L_28_-R_29_-P_30_-G_31_).

**Figure 8:**
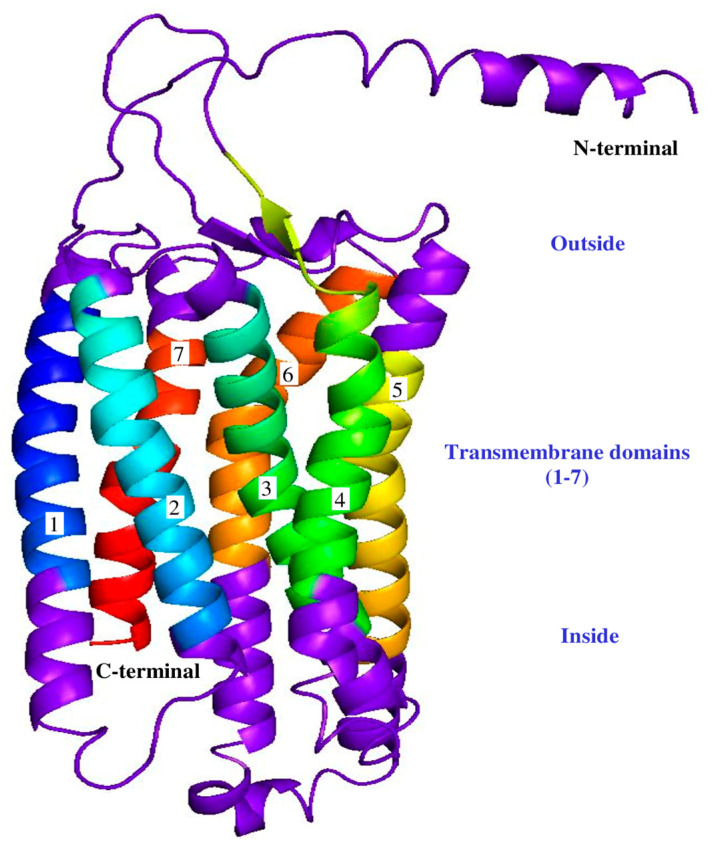
*M. auratus* GnRHR1 protein structure prediction using Robetta and PyMOL software. Three-dimensional structure shows the N-terminal domain, seven (1–7) transmembrane domains, and C-terminal domains. The transmembrane domains are indicated in different colors, while N-terminal and C-terminal domains are shown in purple.

The in silico studies were carried out to understand the structural architecture of the deduced amino acid sequences obtained for *M. auratus* GnRH1/GnRHR1. The three-dimensional structure of the GnRH1/GnRHR1 proteins was established using Robetta and PyMOL software. The analysis predicted that GnRH1/GnRHR1 amino acid sequences have conserved domains characteristic of each peptide family. GnRH1 consists of a signal peptide, a decapeptide, and a GnRH-associated peptide 1 (Figure 7); GnRHR1 peptide shows a transmembrane region with seven domains for GnRHR1, an N-terminal outside region, and a C-terminal inside region (Figure 8).

### 3.5. Transcription Assessment

**Figure 9:**
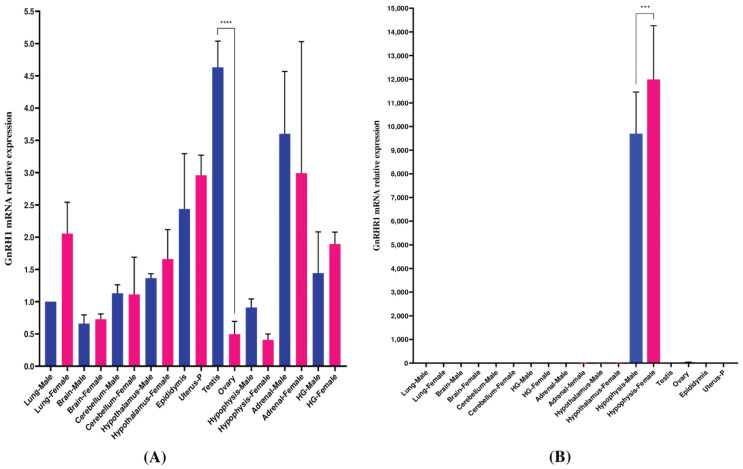
qPCR study of relative gene expression of GnRH1/GnRHR1 from several tissues of *M. auratus*. Relative mRNA abundance of *M. auratus* GnRH1 (**A**) and GnRHR1 (**B**) in adult females (pink bar) and males (blue bar) in several tissues. Vertical bars indicate mean values ± SD of five independent qPCR assays. The statistical difference is shown by one-way ANOVA followed by Dunnett’s post hoc test to compare gene expression between several tissues. Asterisks above the bars indicate significant differences from several tissues. Lung was used as the control (Dunnett’s test, *p* < 0.05). *** and **** indicate *p* < 0.001 and *p* < 0.0001, respectively. *p*-value < 0.05 was considered to be significant.

**Figure 10:**
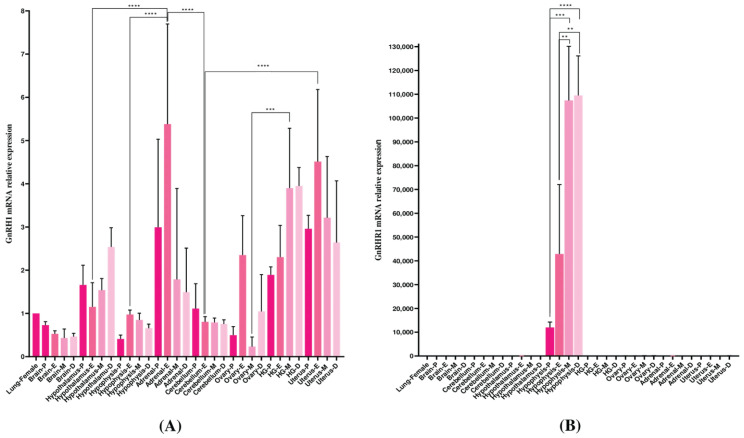
qPCR study of relative gene expression of GnRH1/GnRHR1 from several tissues of female *M. auratus*. Relative mRNA abundance of *M. auratus* GnRH1 (**A**) and GnRHR1 (**B**) in adult females (pink bars) in several tissues during estrous: proestrus (P), estrus (E), metestrus (M), and diestrus (D). P = dark pink, E = medium pink, M = pink, D = light pink. Vertical bars indicate mean values ± SD of five independent qPCR assays. The statistical difference was shown by one-way ANOVA followed by Dunnett’s post hoc test to compare gene expression between several tissues. Asterisks above the bars indicate significant differences from several tissues. Lung was used as the control (Dunnett’s test, *p* < 0.05). **, ***, and **** indicate *p* < 0.01, *p* < 0.001, and *p* < 0.0001, respectively. A *p*-value < 0.05 was considered significant.

The ultimate goal was to assess the expression profiles in *M. auratus* tissues; thus, samples from both male and female sexes were evaluated by qPCR. The GnRH1 expression levels were observed in tissues such as the epididymis, uterus, and testes; transcripts from the hypothalamus, adrenals, and gonads were detected in both sexes. GnRH1 expression was not determined in tissues such as the brain, cerebellum, hypophysis, and ovary (Figure 9A). Transcription levels for GnRHR1 were identified exclusively in the hypophysis of both sexes. GnRHR1 mRNA levels were not detected in tissues such as the brain, cerebellum, gonads, adrenals, hypothalamus, gonads, epididymis, and uterus (Figure 9B). No significant differences were observed in the GnRH1/GnRHR1 transcript expression profiles. Transcript profiles for GnRH1/GnRHR1 were evaluated during the phases of the estrous cycle to measure mRNA levels during the female reproductive cycle. The results indicated differential expression of GnRH1 in the hypothalamus, adrenals, ovary, and uterus (Figure 10A). GnRHR1 levels were higher during metestrus–diestrus in hypophysis and decreased during proestrus–estrus. No GnRHR1 expression was observed in brain, cerebellum, hypothalamus, gonads, ovary, adrenals, or uterus during the estrous cycle (Figure 10B).

## 4. Discussion

The function and regulation of reproductive endocrinology in vertebrates, including humans, is controlled via *GnRH1* and *GnRHR1* genes. Multiple reports, including this one, have indicated that GnRH1/GnRHR1 signaling is the key regulator of HHG axis and reproductive behaviors via synthesis and secretion of gonadotropins, LH, and FSH [23,24,25,26]. The study of GnRH1/GnRHR1 signaling in non-human species is fundamental to generating knowledge at the molecular, evolutionary, biomedical, and biotechnological levels. Previous studies have reported that GnRH1/GnRHR1 signaling in vertebrates contains different molecular variants (GnRH1, GnRH2, and GnRH3; GnRHR1, GnRHR2, and GnRHR3) and tissue expression levels [27,28,29]. Nevertheless, the underlying sex-differentiated molecular mechanism in *M. auratus* induced by the estrous cycle has not yet been completely characterized. Therefore, this study analyzed the molecular structure and transcriptional expression of GnRH1/GnRHR1 signaling in *M. auratus*.

First, this study uncovered the existence of GnRH1 and GnRHR1 peptides in *M. auratus*, previously reported as neuropeptides that regulate reproduction in all vertebrate groups; this suggests a critical ancestral evolutionary relationship [30]. Advances in multi-omics approaches (transcriptomics, proteomics, next-generation sequencing (NGS), and genome-wide association studies (GWAS)) have provided insights into the reproductive endocrinology, but here, molecular techniques such as PCR, RACE, cloning, and Sanger sequencing were used for structural characterization of the coding regions of the *GnRH1* and *GnRHR1* genes from *M. auratus*. These approaches offer precise and detailed determination of nucleotide and amino acid sequences of GnRH1/GnRHR1 peptides associated with reproductive signaling in *M. auratus*, which can be compared with those of various vertebrate species including humans. These results suggest that despite the rise of new technologies, the assays described here remain widely used tools due to several important advantages: high precision, suitability for small or specific sequences, low cost in short projects, lower technical complexity, high reproducibility, visual detection of heterozygous variants, validation of conflicting regions, and clear and easy-to-interpret results.

The predicted amino acid sequences described in these molecular assays include the typical conserved structure of the GnRH1 and GnRHR1 protein families. The amino acid sequence analysis of GnRH1 from the vertebrate group (fish, amphibians, reptiles, birds, and mammals) included here suggests identity and similarity with the signal peptide, the decapeptide, and the GnRH1-associated peptide 1. However, the decapeptide is the only highly conserved sequence—specifically in the amino acids Q_1_, W_3_, S_4_, G_6_, P_9_, and G_10_. Furthermore, phylogenetic analyses grouped and classified the GnRH1 peptide according to its biological classification showing the highest homology percentages in mammals, specifically in the order rodentia. These results support previous domain conservation data and suggest that the GnRH1 peptide has been highly conserved throughout vertebrate evolutionary history. Additionally, its three-dimensional architecture exhibits structural domains like other GnRH1 peptides, thus validating its conformation and structural conservation within the vertebrate group. Similar structural data have been reported for other vertebrate species suggesting its predominant role in reproductive physiology [31,32,33].

The relationship between vertebrate homologs was examined to further understand the origin of the GnRHR1. The predicted amino acid sequence for GnRHR1 from *M. auratus* is 61–92% conserved across anuran and mammalian species. This is nearly identical within the seven putative transmembrane domains. The three-dimensional structure of the *M. auratus* GnRHR1 peptide supports the observations of the transmembrane region. These results suggest very close and specific homology—we hypothesize that mammalian GnRHR1 peptide has a specialized reproductive function in this group of vertebrates. Furthermore, the identification of GnRHR1 structures from anurans suggests the need for further studies to identify the reproductive significance and number of variants in this group of amphibians. Previous studies support the structural findings of the *M. auratus* GnRHR1 peptide: The seven-domain transmembrane structure has been identified in several vertebrate species, suggesting important evolutionary conservation for GnRHR1-mediated signaling and its subsequent regulation of gonadotropins such as LH and FSH [23,25,26,27,29].

This study identified expression in hypothalamic, gonadal, adrenal, epididymal, uterine, and Harderian tissues to evaluate the tissue expression of GnRH1/GnRHR1 signaling in *M. auratus*. Previous reports have indicated that GnRH1 expression depends on development, the endocrine context, and the species. Furthermore, GnRH1 expression has been described as primarily localized in hypothalamic neurons; however, it has also been detected in several brain nuclei and gonads of some species [34,35,36]. Hypothalamic tissue expression studies suggest its classical involvement and regulation in the HHG axis; however, its localization in extrahypothalamic tissues could suggest local autocrine and paracrine regulation. Furthermore, GnRHR1 expression varies according to species, physiological state, and tissue. The highest and most functional expression of GnRHR1 is found in the anterior hypophysis or adenohypophysis, specifically in gonadotropic cells, where it regulates the synthesis and secretion of LH and FSH [37,38,39]. *M. auratus* GnRHR1 expression could not be detected in other tissues and was exclusively expressed in hypophysis with variations during the phases of the estrous cycle, suggesting its probable regulation by sex steroids. The results suggest that the hamster could be considered a standard model for studying the regulation of the HHG axis. Previous studies using the hamster model and its regulation by INSL3/RXFP2 could support this concept and the notion that the hamster could help explain reproduction [17].

This study does have some limitations. The hamster is a common model in neuroendocrine and reproductive studies, but results from hamsters should be interpreted with caution due to the seasonal regulation of GnRH1 and GnRHR1, the limited availability of molecular tools (such as protein detection and localization by Western blot, immunohistochemistry, in situ hybridization, and transcriptomic techniques such as RNA-Seq), and species-specific physiological differences.

## 5. Conclusions

In summary, these findings might provide scientific evidence of using the hamster as a biological model—especially in reproductive, neuroendocrine, and comparative research related to GnRH1/GnRHR1 signaling. Although evolutionary differences exist, the sequence and function of GnRH1/GnRHR1 are highly conserved in vertebrates—particularly in mammals. The results can be extrapolated to humans in areas such as endocrine infertility, hormonal regulation, and therapies focused on GnRH1/GnRHR1 agonists and antagonists. The evidence in this research confirms that *M. auratus* GnRH1/GnRHR1 signaling has a wider tissue distribution than originally thought. Its dynamic expression, dependent on the physiological and hormonal context, suggests that it fulfils functions complementary to its classic role in reproduction and hormonal regulation.

## Data Availability

Data are contained within the manuscript.
